# The role of dendritic cells in the immunomodulation to implanted biomaterials

**DOI:** 10.1038/s41368-022-00203-2

**Published:** 2022-11-04

**Authors:** Siyuan Wang, Yanqi Chen, Zhaoting Ling, Jia Li, Jun Hu, Fuming He, Qianming Chen

**Affiliations:** Stomatology Hospital, School of Stomatology, Zhejiang University School of Medicine, Clinical Research Center for Oral Disease of Zhejiang Province, Key Laboratory of Oral Biomedical Research of Zhejiang Province, Cancer Center of Zhejiang University, Hangzhou, 310006 China

**Keywords:** Biophysical chemistry, Biomedical materials, Biomaterials - cells

## Abstract

Considering the substantial role played by dendritic cells (DCs) in the immune system to bridge innate and adaptive immunity, studies on DC-mediated immunity toward biomaterials principally center on their adjuvant effects in facilitating the adaptive immunity of codelivered antigens. However, the effect of the intrinsic properties of biomaterials on dendritic cells has not been clarified. Recently, researchers have begun to investigate and found that biomaterials that are nonadjuvant could also regulate the immune function of DCs and thus affect subsequent tissue regeneration. In the case of proteins adsorbed onto biomaterial surfaces, their intrinsic properties can direct their orientation and conformation, forming “biomaterial-associated molecular patterns (BAMPs)”. Thus, in this review, we focused on the intrinsic physiochemical properties of biomaterials in the absence of antigens that affect DC immune function and summarized the underlying signaling pathways. Moreover, we preliminarily clarified the specific composition of BAMPs and the interplay between some key molecules and DCs, such as heat shock proteins (HSPs) and high mobility group box 1 (HMGB1). This review provides a new direction for future biomaterial design, through which modulation of host immune responses is applicable to tissue engineering and immunotherapy.

## Introduction

**Fig. 1:**
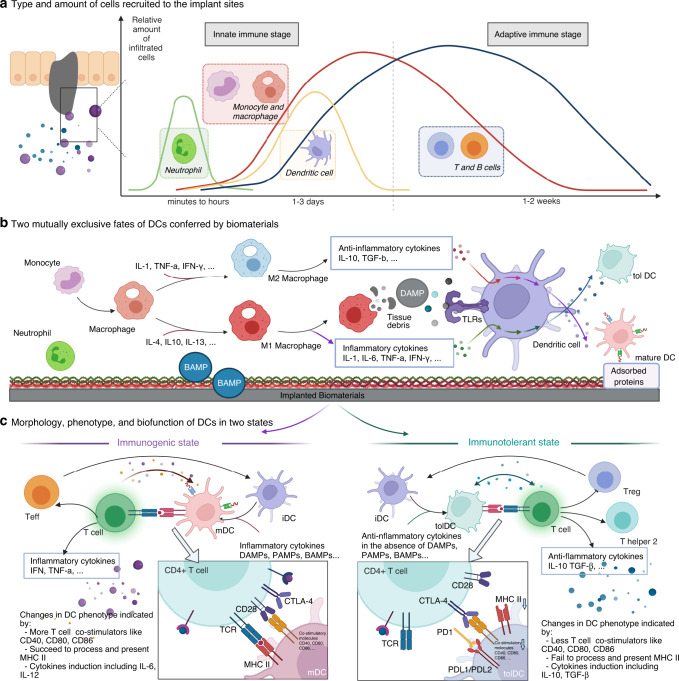
Important immune events involved in host response towards implanted biomaterials. **a** Host proteins majorly adhere to the surface of biomaterials within 4 h, which are followed by a series of subsequential chain responses like the infiltration of immune cells consecutively (see Supplementary Fig. S1 for detailed illustration). **b** DCs surveying in the periphery signal foreign biomaterials through BAMP. Meanwhile, driven by cytokines and chemokines released from the surrounding lymphocytes, DCs undergo the transformation towards mDCs or tolDCs. **c** Specifically, having recognized the biomaterial through BAMP, iDCs experience the shift towards antigen-capturing mDCs with a ‘stellate’ morphology and upregulated expression of stimulatory and co-stimulatory molecules such as MHC-II, CD80, and CD86, which further initiated T cell-mediated adaptive immune response. On the contrary, iDCs encountering self-antigens or in the absence of foreign signals would differentiate into tolDCs, which downregulate the expression of co-stimulatory molecules, enhance the expression of inhibitory molecules, and promote anti-inflammatory cytokines TGF-β and IL-10. *The arrows with solid lines in two different colors both indicate a progressive meaning, with the red arrow involved in inflammation and the blue one in immunomodulation. In addition, the double-ended arrow in the right part of Fig. 1(**c**) indicates that productions from T cell could also induce tolDCs differentiation (e.g., TGF-β, IL-10)

Biomaterials and biomedical devices implanted in a host trigger a series of orchestrated immune reactions, including blood-biomaterial contact, the formation of adsorbed proteins, and recruitment of immune cells (Fig. 1a, b).^1^ Successful integration of implanted biomaterials requires complete removal of inflammation and tissue regeneration, which is highly related to complex immunity. In this context, ongoing efforts have been devoted to excavating the interplay of biomaterials and host immune systems, the involved signaling molecules, and the underlying mechanisms.^2^ Biologically distinct subsets of DCs originate from hematopoietic stem cells (HSCs) in the bone marrow and regulate the function of T cells in different ways,^3^ including myeloid/conventional DCs, plasmatic DCs, and follicular DCs.^4–6^ As the most efficient antigen-presenting cells (APCs), they serve as a jointing band between innate and adaptive immunity and maintain a core position in the process of injury and healing. Recent knockout studies have identified the critical role of DCs in immune responses.^7^ For instance, conditional and constitutive ablation of DCs in mice might lead to spontaneous development of autoimmune disease.^8^

Under steady state, DCs surveying in the periphery signal foreign antigens through pathogen-associated molecular patterns (PAMPs) or damage-associated molecular patterns (DAMPs), thus generating a battery of immune reactions.^9,10^ During the process, pattern-recognizing receptors (PRRs) are adopted by immature tissue-resident DCs (iDCs) to capture antigens, including Toll-like receptors (TLRs), C-type lectin receptors (CLRs) and nucleotide-binding oligomerization domain (NOD)-like receptors (NLRs).^10^ Initiated by foreign signals, iDCs are transferred into mature DCs (mDCs), which are capable of launching T-cell mediated immune responses against foreign attacks.^11^ iDCs encountering self-antigens or in the absence of foreign signals differentiate into tolerogenic DCs (tolDCs), which promote immune tolerance.^12^ The double-edged role of DCs seems to have been extensively studied for orchestrating immune responses,^1^ of which plasticity is generated from their unique role as sentinels, consecutively signaling their peripheral cues and exerting context-dependent properties.^13,14^ Considering this, recent studies have revealed that modifying the physiochemical properties of implanted biomaterials would generate exceptionally different immune milieus and elicit varied DC-mediated host responses,^15^ thus leading to immune responses or tolerance.^16–18^ For instance, genetically modified^19–21^ or pharmacologically conditioned^22–24^ maturation-resistant DCs have been introduced to promote allograft survival, tissue regeneration, vaccine delivery,^25^ autoimmune disease treatment^26^, and tumor immunotherapies. Therefore, a comprehensive elucidation of the interplay between biomaterial physiochemical properties and DC phenotypes is truly important.^27^ Based on this, future efforts might be attributed to inducing tolerogenic, ‘alternatively activated’, and effective mDCs via biomaterial surface modifications in an attempt to disseminate the profound application of biomaterials.^28,29^ Nevertheless, within the limit of our knowledge, approaches to modulate the function of DCs have historically focused on inducing the bioinertness of the materials but overlook their intrinsic bioactivity, which is critical for tissue regeneration.^30^ Moreover, the underlying mechanisms involved in DC-biomaterial interactions have not been well established yet. In particular, the definition and connotation of BAMP has been hardly expounded upon, much less the idiographic chemical composition and structures. Herein, we provide an in-depth look at the current knowledge on the multifaceted effects of the physiochemical properties of biomaterials on DC biofunction and explicate DC behaviors toward implanted biomaterials, with a specific emphasis on the underlying mechanisms. We believe that understanding this complex scenario will assist in the innovative design and clinical application of biomaterials with desired immune effects in tumor therapy, vaccine delivery, and tissue engineering.

## Host response to implanted biomaterials

Biomedical devices or materials implanted in a host universally trigger a highly orchestrated chain of events, including inflammation, and the balance of the immune system breaks down when inflammation is excessive or becomes chronic.^31,32^ When implanted into the host, biomaterial comes into contact with blood or humoral fluids, and proteins will instantly adsorb to the surface of the material.^1^ Generally, host proteins primarily adhere to the surface within 4 h, which is followed by a series of subsequent chain responses occurring on the surface of the biomaterial, including complement activation, the coagulation cascade, and immune cell adhesion.^1^ Early infiltration of the neutrophil population was observed in minutes to hours according to Leifer,^32^ which lasted until 24–48 h after implantation and was followed by monocyte accumulation on biomaterials as well as differentiation into macrophages by 72 h postimplantation.^33–35^

With a relatively limited proportion of approximately 0.5%–1.5% of the total mononuclear cells in peripheral blood, as well as short lifetimes of days to weeks,^34,36,37^ DCs serve as the center of the immune system, providing a vital link between innate and adaptive immune responses.^10,38,39^ In contact with biomaterials, iDCs experience a shift toward antigen-capturing mDCs (Fig. 1c), which are characterized by a ‘stellate’ morphology composed of actin.^40^ Apart from this, many coordinated events are involved in DC maturation, including the upregulated expression of peptide-major histocompatibility (MHC)-I and -II complexes permitting effective antigen presentation, enhanced expression of adhesion molecules and chemokine receptors, and prominent allo-stimulatory capacity and pro-inflammatory cytokine secretion profiles, including IL-1β, IL-6, and IL-12p70.^10,41–43^ As evidenced by Yoshida, markedly increased presentation of costimulatory molecules, including CD80, CD83, and CD86, was observed at early time points (6 h, 24 h) on DCs treated with 75:25 poly(lactic-co-glycolic acid) (PLGA) films and MPs.^44^ As a result of the development of a repertoire of chemokine receptors, including CCR7,^45,46^ mDCs spontaneously migrate to local lymph nodes.^11^ Subsequently, T-helper cells were spotted as early as the third day locally at the implantation sites and in the contralateral bone marrow according to a mouse model, signaling the arousal of adaptive immune responses, which lasted over seven days.^47^ DC-mediated T-cell activation depends on three major signals: (i) the first signal refers to the combination of MHC-peptide complexes with antigen-specific TCRs; (ii) recognition and involvement of costimulatory molecules exhibited on DCs, including CD80 and CD86, by their receptors on the surface of T cells (e.g., CD28, CD40, CTLA-4), which constitutes the second signal; (iii) other soluble or membrane-bound signals, such as IL-12 and type I interferon (IFN-I), consisting of a “polarizing” signal.^48^

In contrast, iDCs encountering certain biomaterial signals, which have not been fully elaborated, differentiate into tolDCs.^12^ TolDCs preserve the ability of antigen presentation to antigen-specific T cells while downregulating the expression of costimulatory molecules (e.g., CD80, CD86, CD40) or proinflammatory cytokines, including IL-12, and enhancing the expression of coinhibitory molecules (e.g., IgG-like transcript 3, PDL1) and anti-inflammatory cytokines, such as transforming growth factor-β (TGF-β) and interleukin-10 (IL-10)^49–54^ (Fig. 1c). Immunological tolerance was maintained by inducing T-cell apoptosis, unresponsive T-cell responses, Treg cell production and inhibition of T-cell responses.^8,9,27,55–57^

## Biomaterials properties modulating DC response

**Fig. 2:**
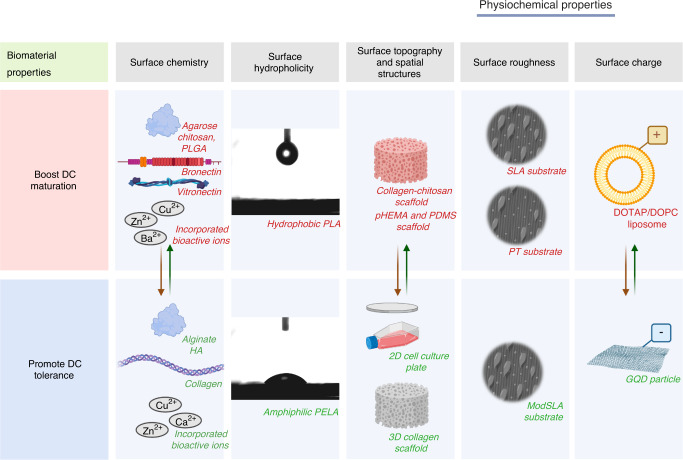
Biomaterials properties modulating DC response. Effects of physiochemical properties of biomaterials that potentially influence DC phenotype and function are summarized in five columns: surface chemistry, hydrophilicity, topography and spatial structures, roughness, and surface charge. Conjugated peptides, chemical moieties, and elemental concentration can surely determine DCs behavior, and DCs seeded on agarose, chitosan or PLGA would boost DC maturation, whereas those treated with alginate or HA were associated with immune-tolerance. Furthermore, PLA with higher hydrophobicity than PELA could promoted effective antigen uptake and induced a higher level of DC maturation. However, DCs seeded on 3D scaffolds can either mature increasingly (e.g., collagen-chitosan scaffolds, pHEMA, and PDMS) or decreasingly (e.g., 3D collagen microenvironment) in comparison with those cultured on 2D culture plates. DCs seeded on titanium substrates with similar surface roughness (SLA, modSLA) presented different maturation. DOTAP/DOPC liposome, with a relatively cationic charge, potently promoted DC maturation, while negatively charged GQD could significantly impair DC-mediated immune reactions. Notably, the paired arrows with two directions in the figure indicated that contrasting outcomes were reported in terms of their effects on DC activation, and no definitive judgment could thus far be drawn. Specifically, these material properties may interact synergistically with each other to yield a microenvironment harmful to or favorable for tissue repair and regeneration. *Abbreviations: PLA poly(D,L-lactic acid), PELA poly(monomethoxypolyethylene glycol-co-D,L-lactide), pHEMA poly(2-hydroxyethyl methacrylate), PDMS poly(dimethylsiloxane), GQD graphene quantum dot

Modulating the immune-inflammatory axis via biomaterials can significantly ameliorate the efficacy of tissue engineering.^58^ Recently, an increasing number of studies have incorporated biomedical polymers with immunogenic or immunomodulatory molecules in an effort to regulate host responses to implanted biomaterials. Nevertheless, the influence of the intrinsic physiochemical properties of implanted biomaterials on DC phenotype and function has been overlooked. A better understanding of this complex interplay will help to modify the particular immune function while eliminating unexpected immune reactions.^59^ To date, many studies have focused on specific material properties, including chemical composition, surface chemistry, hydrophilicity, topography and roughness, spatial structures, surface charge, and incorporated bioactive inorganic ions, which are comprehensively characterized and discussed separately in this section and are summarized in Table S1 and Fig. 2.

### Surface chemistrypatibility and tissue regeneration

Biomaterial-induced immune responses are directly determined by the surface chemistry, such as conjugated peptides or chemical moieties, as well as elemental concentrations of carbon, oxygen, and nitrogen.^60^ Organic biomaterials, including agarose, alginate, chitosan, hyaluronic acid (HA), and 75:25 PLGA, are commonly applied in tissue engineering^61^ and display immunologic roles in the microenvironment.^62^ Yoshida et al. explored the effects of agarose and 75:25 PLGA MPs and films on human monocyte-derived DCs^63^ and murine bone marrow-derived DCs.^44^ They reported that under treatment with PLGA and agarose MPs or films, DCs, regardless of cell origin, showed an upregulation in expression of MHC II and costimulatory molecules, such as CD80 and CD86, in contrast to iDCs but to a lesser extent than that of lipopolysaccharide (LPS)-induced DCs. Babensee generated biofilms of five materials (agarose, alginate, chitosan, HA, PLGA) by casting techniques and examined their influences on iDCs derived from human peripheral blood monocytes.^62^ They found that chitosan and PLGA (and agarose to a lower level) films could promote DC costimulatory capacity equivalent to LPS-induced DCs, whereas alginate or HA films inhibited the costimulatory expression of DCs. Similarly, Park et al. found that cultivating DCs with alginate and HA did not stimulate DCs, while DCs seeded on PLGA or chitosan films presented upregulated surface maturation molecules, including CD80, CD86, CD83, and human leukocyte antigen-DQ (HLA-DQ), accompanied by enhanced proinflammatory cytokine secretion, such as tumor necrosis factor-α (TNF-α) and allo-stimulatory T-cell proliferation, as confirmed via mixed lymphocyte reactions (MLR).^61^ Concludingly, diverse biomaterial chemistries might contribute to the development of distinct BAMPs in terms of proteins and carbohydrates.

Compounding this, DC morphology and release of cytokines such as IL-12p40 and IL-10, are modulated via adhesive substrates-associated signaling.^64^ Furthermore, Archaya et al. confirmed the substrate-dependent differences in the stimulatory and costimulatory capacities of DCs derived from nonobese diabetic mice.^65^ Among adhesive substrates, including fibronectin, fibrinogen (Fg), collagen, vitronectin, laminin, albumin and serum, DCs cultivated on vitronectin induced the highest immunogenicity, collagen elicited the highest IL-10 secretion in DCs, and the fibronectin-coated surface gave rise to the highest level of maturation in T cells.^66^ However, the expression of MHC-II and CD40, cytokine secretion profiles, and capability of inducing allogeneic T-cell proliferation of DCs were independent of pre-adsorbed adhesive proteins.^67^ The seemingly contrasting outcomes might result from the solid substrates (Bioflex plates), which were different in nature from the TCPs used in prior studies as indicated by the authors.

In terms of biomaterial surface modification, incorporation with bioactive inorganic ions is universally viewed as a convenient, effective, and long-lasting tool to promote desired effects.^68^ For instance, zinc has displayed superior antibacterial and osteogenic properties in previous investigations, and its role is irreplaceable in immune responses.^69–71^ Specifically, the exogenous addition of Zn^2+^ induced a tolerogenic phenotype of bone marrow-derived DCs, with suppressed expression of MHC II and elevated expression of PD-L1 and PD-L2, skewing the balance of Treg/Th17 cells in favor of FoxP3^+^ Tregs in a model of Histoplasma capsulatum fungal infection.^72^ Meanwhile, Chan et al. reported that in comparison with alginate polymer-treated DCs, those encapsulated in Ca^2+^-crosslinked alginate presented higher levels of CD86 and MHC II while producing more inflammatory cytokine IL-1β, indicative of higher maturation levels, while Ba^2+^-crosslinked alginate had no such effect. Additionally, significantly upregulated IL-1β secretion was observed from calcium alginate gel-surrounded tissue in mice.^73^ Moreover, Schuhladen and coworkers incorporated bioactive glasses (BGs) with biologically active ions, Cu^2+^, Zn^2+^, and Cu-Zn^2+^, on DCs and reported that the presence of copper modulated the phenotypic performance of tolDCs in favor of T-cell polarization into an anti-inflammatory Treg milieu.^74^ In contrast, Li et al. proposed that γ-AlOOH mesostrands incorporated with hierarchical Cu- and Zn-buds (Cu- and Zn-AMSs) promoted BMDC maturation and proinflammatory cytokine release, which further facilitated T-cell proliferation in mice.^75^ Heterogeneities within bioactive ion concentrations among different types of biomaterial carriers might be plausible for the different outcomes of DC behaviors. In this context, increasing research efforts should focus on incorporating available biomaterials with biological ions in an effort to improve the effectiveness and security of their profound applications.

### Surface hydrophilicity and hydrophobicity

Hydrophobicity or the hydrophobic portion of biomaterials is their inherent immunogenicity, which serves as a universal language of injury and repair.^76^ In contrast, hydrophilic surfaces similar to those present in natural tissue may be able to stimulate immune cells to switch activation, thus preventing a chronic immune response.^77^ Specifically, the wettability of the biomaterial surface determines the proteins adsorbed to it and the subsequent formation of blood clots and fibrin networks.^78^ However, protein adsorption on a hydrophobic surface alters its conformation through hydrophobic interactions, exposing the intrinsic domain and promoting the recruitment of host immune cells.^79,80^ Yang et al. fabricated a novel superamphiphilic material polycaprolactone (PCL) with an interconnected hierarchical pore structure and ascendant wettability, as confirmed by a water contact angle of 45.3°. The hydrophilic PCL displayed a facilitating effect on DC adhesion and proliferation when compared with hydrophobic surfaces. However, the authors did not further investigate the phenotype and function of DCs.^81^ Meanwhile, Liu et al. confirmed that poly(D,L-lactic acid) (PLA) spherical microparticles^19^ possessed similar size, charge, and morphology but higher hydrophobicity than PLGA or poly(monomethoxypolyethylene glycol-co-D,L-lactide) (PELA), which promoted effective antigen uptake and induced a higher level of DC maturation. In addition, adhesion force measurements further validated the strong interaction between highly hydrophobic PLA MPs and cell membranes, favoring MP internalization and the subsequent elicitation of immunity.^82^ Chaudhary et al. also underscored that hydrophilic surfaces preferentially modulate biological functions, including protein adsorption, cell attachment, and proliferation, thereby enhancing bioactivity and regulating biological functions.^83^ By immobilizing the adsorbed proteins, it is therefore promising to develop a biointerface via improvement in hydrophilicity that can promote biocompatibility and tissue regeneration.^58^

### Surface topography, and spatial structures

Surface attributes such as porosity, roughness, three-dimensional structures, and the presence and type of micro- and nano-structures (NS) have profound influence on the phenotype and function of immune cells.^42^ Micro- and nano-structures have been shown to significantly affect DC behaviors.^58,78^ Compounding this, DCs seeded on poly(2-hydroxyethyl methacrylate) (pHEMA) and poly(dimethylsiloxane) (PDMS) scaffolds matured increasingly with decreasing pore size, with those cultivated on 20-μm pore scaffolds matured most remarkably.^84^ In this context, topochip, which was fabricated with polylactic acid (PLLA) or polyethylene oxide/polybutyl terephthalate (PEO/PBT), has been introduced into biomedical immunity, thereby enabling the screening of DC response to certain important biomaterial properties in a high-throughput manner.^85^ For instance, Unadkat et al. developed chips of PLGA with 2176 different, nonbiased, random surface patterns based on mathematical algorithms.^86^ Using this topochip, Kou et al. reported that a human DC-like cell line (KG-1 cells) adhered discordantly toward different topoUnits.^87^ Collectively, these findings highlighted that the surface topographic characteristics of biomaterials are of great significance to DC phenotype and functions and that it is beneficial to interfere with cell-biomaterial interactions by modifying surface topography in an effort to manipulate the tissue repair responses.

With most of the current studies ongoing relative to the biomaterial-surrounding milieu, the three-dimensional (3D) spatial structure, however, might better depict the microenvironment where DCs function.^88^ In fact, good cytocompatibility of 3D hydrogels at various concentrations was shown in terms of DC extension and surface marker expression.^89^ Koen van den Dries et al. reported that 3D micropatterned surfaces inhibit PGE2-mediated RhoA activation, which resulted in impaired podosome dissolution and higher MHC-II expression of DCs, suggesting the important role of three-dimensional geometry cues in regulating DC adhesive and immunomodulatory properties.^90^ Additionally, Daneshmandi et al. developed collagen-chitosan scaffolds to mimic the 3D microenvironment for DCs, where higher levels of maturation and cytokine production, including IL-6, IL-12, and TNF-α, were observed in comparison with those cultured on 2D culture plates. Furthermore, the induced secretion of IFN-γ and IL-4, together with the downregulated release of TGF-β, was detected during DC-T-lymphocyte cocultures in a 3D system.^88^ Consistently, Chen et al. reported that after exposure to pHEMA and PDMS scaffolds, the secretion of MIP-1α, IL-6 and TNF-α, along with CD86 expression on DCs, significantly increased when compared to those grown on NTCPs, indicating promotion of mDC-induced inflammation.^84^ Nevertheless, Sapudom et al. showed that predifferentiated mDCs presented higher levels of CCR7, MHC II, CD80, and CD86 when seeded on 2D tissue culture plastic, which were attenuated by a 3D collagen microenvironment.^91^ The deviations in DC maturation and function might be attributed to different cell lines as well as the intrinsic hydrophilicity and surface chemistry of the biomaterials adopted. Altogether, spatial structures may in this manner interact synergistically with other properties to yield a microenvironment favorable for tissue repair and regeneration.

### Surface roughness

Roughness is another critical parameter determining immune responses, particularly for metallic implant materials. Nevertheless, it has been reported that among the multiplex properties that influence DC behavior, surface roughness might not be the critical determinant. Titanium substrates have been fabricated with typical microtopography as well as surface energy by Kou and coworkers:^16^ PT discs were chemically polished to be finely smooth, SLA substrates displayed a hierarchical structure of cavities with 10–50 µm indentations completely superposed by 1–2 µm pores,^92^ and modSLA surfaces displayed similar roughness as SLA while maintaining its high surface energy. PT- and SLA-treated DCs both displayed indistinguishably high dendritic morphology and expressed higher levels of CD86 than those cultured on TCPs. ModSLA surfaces, on the other hand, rendered DCs cultivated on them to maintain a CD86 expression level similar to iDCs and process-free immature morphology.^16–18^ Enhanced expression of proinflammatory IL-6, IL-12, and IL-18 was detected in DCs cultured on PT or SLA surfaces, while IL-1ra, IL- 4, IL-10, and TNF-α were reduced, and the outcome was the opposite in terms of modSLA surfaces.^18^ The characteristics of biomaterial surfaces were confirmed by assessment of surface roughness, contact angle, and surface chemistry via XPS analysis, as listed in Table 1.^16–18,77,93–97^ Collectively, different behaviors of DCs might be interpreted as the outcome of a higher oxygen percentage and hydrophilicity, whereas current studies have concluded that surface roughness was not decisive in DC-biomaterial interactions.^17^ Nevertheless, explicit effects of surface roughness on DC phenotype and function are not fully elaborated, and this prospective should be generalized in future efforts.

**Table 1:** Physiochemical properties of Titanium surface

Physiochemical properties		PT	SLA	modSLA	Ref.
Roughness (Ra)/μm		0.59 ± 0.019	3.58 ± 0.042	3.64 ± 0.029	^77^
		0.38 ± 0.02	1.78 ± 0.02	1.78 ± 0.04	^17^
		~1.8	250–500	~0.5	^96^
Air-water contact angle		93.6°	120.9°	0°	^77^
		~90°	~130°	~0°	^96^
		(91.31 ± 7.30)°	(139.88 ± 8.69)°	0°	^92^
		Not mentioned.	(138.3 ± 4.2)°	0°	^93^
		Not mentioned.	(125.9 ± 6.8)°	0°	^78^
Surface Chemistry	**O/%**	48	48	60	^77^
		47.6 ± 1.2	45.7 ± 1.12	50.58 ± 2.03	^17^
		Not mentioned.	50.2 ± 2.6	60.1 ± 0.7	^94^
		Not mentioned.	53.21	36.77	^96^
		46.8 ± 1.9	50.2 ± 2.6	60.1 ± 0.7	^95^
		Not mentioned.	49.2 ± 2.1	61.1 ± 0.9	^92^
		Not mentioned.	44.2 ± 1.9	55.0 ± 2.0	^93^
	**Ti/%**	18	15	22	
		13.48 ± 0.49	13.75 ± 1.84	15.17 ± 0.56	
		17.9 ± 1.0	14.3 ± 1.4	23.0 ± 1.1	
		Not mentioned.	19.33	11.23	
		Not mentioned.	14.3 ± 1.4	23.0 ± 1.1	
		18.4 ± 1.6	14.3 ± 1.3	22.5 ± 0.9	
		Not mentioned.	18.4 ± 1.6	26.5 ± 0.9	
	**N/%**	1.78 ± 0.14	1.37 ± 0.13	1.51 ± 0.14	
		1.2 ± 0.4	1.3 ± 0.3	0.7 ± 0.2	
		Not mentioned.	1.12	0.76	
		Not mentioned.	1.3 ± 0.3	0.7 ± 0.2	
		0.6 ± 0.2	1.3 ± 0.4	0.7 ± 0.3	
	**C/%**	31	35	17	
		40.86 ± 1.47	39.19 ± 1.95	32.74 ± 1.60	
		29.2 ± 1.5	34.2 ± 2.0	14.9 ± 0.9	
		Not mentioned.	26.35	26.20	
		Not mentioned.	34.2 ± 2.0	14.9 ± 0.9	
		30.9 ± 2.1	35.2 ± 2.2	14.2 ± 1.2	
		Not mentioned.	37.3 ± 3.4	18.4 ± 2.7	
Topography		Micron and submicron structures	Micron structures	Micron and nanostructures	^77^
		Not mentioned.	Macro and microstructure without characteristic NS.	Characteristic NS with needle-like shape of the crystallites with dimensions of about 10 nm in width and 30 nm in length.	^78^
Protein adsorption		Not mentioned.	Fg: 525.2 ± 135.2 Fn: 331.9 ± 134.8	Fg: 4 969.9 ± 1 619.5 Fn: 3 835.8 ± 795.6	^78^

The previously characterized surface properties of the Titanium discs were summarized in Table 1 for mean-peak-to-valley roughness (Ra), air-water contact angle, surface chemistry, topography, and protein adsorption

### Surface charge

According to Gammon, the surface charge on the biomaterial surface is critical in modulating the migration and differentiation of immune cells and is generalizable across different biomaterials.^59^ According to Andorko et al., neutral to positive zeta potentials would induce conspicuous DC activation.^98^ For instance, Ma et al. evaluated DOTAP/DOPC liposome-regulated immune responses and reported that liposomes with a relatively cationic charge potently promoted DC maturation and antigen uptake, whereas low-charge liposomes failed to facilitate DC-mediated immunity even at high concentrations.^99^ Meanwhile, with their negative surface charge, graphene quantum dot (GQD) particles (large GQD: (−9.0 ± 1.5) mV; small GQD: (−9.4 ± 0.8) mV) significantly impaired DC-mediated allogeneic T-cell proliferation and Th1/Th17 polarization, which was in accordance with the modified secretion of IL-12p70 and altered expression of CD40, CD83, and CD86.^100,101^ Nevertheless, cationic materials might, on the other hand, lead to diminished activation of DCs with suppressed antigen uptake.^98,102,103^ In fact, investigations on the role of surface charge in DC-biomaterial interactions are ongoing, and the aforementioned attempts primarily suggest that the surface charge of biomaterials is critical in DC activation and is highly manageable, which might be a future direction in bioengineering and vaccine delivery.^104–106^

Biomedical strategies targeting DCs for modulating the balance between immunomodulation and immune reactions can significantly improve the efficacy of tissue engineering. Harnessing the surface properties (surface chemistry, hydrophilicity, topography and spatial structures, roughness, and surface charge) of biomaterials are promising to modify immune reactions while eliminating unexpected adverse reactions. Nevertheless, materials properties are generally intricate and interlace with each other, rendering what and how biomaterial properties that potentially govern DC-mediated immune responses unknown. For instance, interpretation of the varied behaviors of DCs in response towards the five commonly adopted biomaterials (agarose, alginate, chitosan, HA, and PLGA) triggered big confusions. Park indicated that hydrophobic PLGA and chitosan with highly cationic glucosamine displayed higher levels of maturation. Nevertheless, alginate, absent in hydrophobicity or negative charges, also induced higher levels of maturation within DCs in terms of TNF-α and IL-6 release.^107^ It is thereby reasonable to preliminarily postulate that apart from hydrophobicity and cationic charges associated with the biomaterial surface, the inherent chemical compositions, such as carbohydrate units contained in mannuronic acids and guluronic acids, might possibly determine the resultant phenotype of DCs upon biomaterial exposure. In addition, carbon dioxide and hydrocarbon contaminants introduced from the environment during film formation, and rearrangement of hydrophobic sites of biomaterial molecules during biomaterial processing procedures might, to some degree, account for the modified DC behaviors.^61^ To cope with the limitations in delineate the interplay between single material property and DC performance, Kou et al. performed principal component analysis (PCA), and demonstrated that titanium surface hydrophilicity and concentration of oxygen were associated with anti-inflammatory immature phenotype of DCs. Considering that these biomaterials of different forms have been ubiquitously adopted as scaffolds or cell carriers for tissue engineering,^108–110^ persistent efforts should be distributed here to delineate a comprehensive map with regard to material property-DC phenotype interactions. Fortunately, the application of immune-mediated tissue engineering has witnessed immense growth in recent decades with the development of high-throughput strategies (e.g., PCA and partial least squares regression (PLSR)).

## Biomaterial-associated molecule patterns

**Fig. 3:**
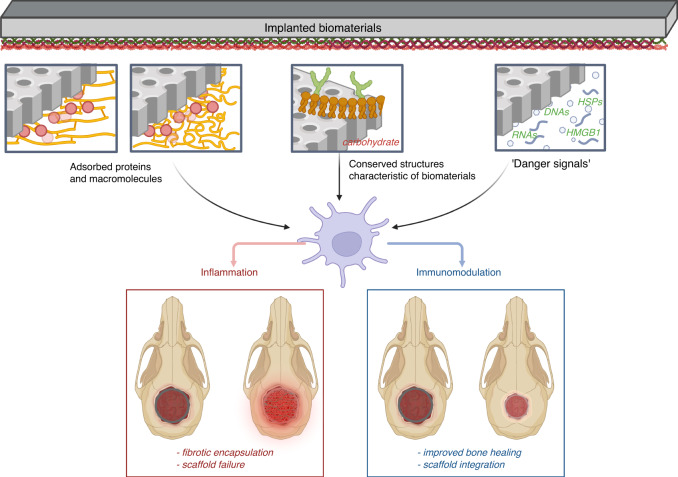
A brief illustration of Biomaterial-associated molecular patterns. When implanted in the host, biomaterials are capable of adsorbing or desorbing contacting proteins and form a layer of adsorbed protein. Biomaterial surface properties determine the adsorbed proteomic profiles and the subsequential cellular interactions. These recognizable motifs are referred to as ‘biomaterial associated molecule patterns’, and the connotation of which could be could be summarized as three main parts: adsorbed proteins which can be allogenic (derived from materials or contamination) or autologous (ECM existed in blood or body fluids); soluble ‘danger signals’ (e.g., DNA, RNA, HMGB1, HSOs); and characteristics of the material itself

When implanted in the host, biomaterials are capable of adsorbing or desorbing contacting proteins and form a dynamic layer of adsorbed protein, thus encouraging DCs binding and tissue repair.^111,112^ This layer of adhesion protein can be allogenic (derived from materials or contamination) or autologous (extra-cellular matrix (ECM) existed in blood or body fluids), and modulate host responses, such as coagulation, complement activation, and innate immunity.^29,113^ A recent research uncovered that biomaterial surface properties determined the adsorbed proteomic profiles and the subsequential cellular interactions.^114^ These recognizable motifs are referred to as ‘biomaterial associated molecule patterns’ (BAMP), which are similar with DAMP/PAMP. As first put forward by Babensee, BAMP could be interpreted as either cognate binding ligands relevant to adsorbed proteins, soluble ‘danger signals’ such as DNA, RNA, HSPs, and HMGB1, as well as intrinsic structures characteristic of biomaterials, in particular carbohydrates.^115–117^ However, the absolute conformational structures and chemical composition of BAMP have not been fully clarified. Herein, we elaborate on the connotation of BAMPs based on related studies and enumerate several BAMPs that are critical in DC-biomaterial interactions (Fig. 3).

### Adsorbed proteins and other macromolecules

A battery of studies has confirmed activation of DCs under exposure towards HA, up-regulated adherence to fibronectin, and induced mature phenotype treated with type 1 collagen and fibronectin.^118^ In addition, adhesion-limiting proteins (albumin, vitronectin, and fibronectin) have higher affinity to hydrophilic surfaces, whereas adhesion-promoting proteins (fibrinogen and IgG2) adhere easily to hydrophobic surfaces.^1^ Acharya et al. proposed that DC morphology and inflammatory cytokine expression (IL-12p40 and IL-10), instead of adhesion or expression of costimulatory molecules are adhesive substrate-dependent.^64^ Nevertheless, Lewis reported that neither surface expression of stimulatory molecules or cytokine secretion profiles were modulated via adhesive proteins, regardless of laminin, collagen, or fibrinogen.^67^ These seemingly contrasted results may be attributed to the different time points the two experiments undertake (24 h, 1 h).^28^ Compounding this, osteopontin (OPN),^119^ vitronectin,^120^ and fibronectin^121^ adsorbed to foreign biomaterials may be recognized by DCs and resulted in foreign body reactions (FBR).

Furthermore, Escalante and Swartzlander^122,123^ clarified the protein adsorption profiles on the surface of PEG hydrogels via liquid chromatography-tandem mass spectrometry. According to them, both regulators of wound healing, such as alpha-2 macroglobulin and apolipoprotein A-I, together with inflammation stimulators, including vitamin D binding protein and complement component 3 (C3), were identified at the interface. Proteins containing the bioactive motif Arg-Gly-Asp (RGD) serve as the primary cell attachment sites for a battery of adhesive ECM proteins.^124,125^ Identified on fibronectin,^126,127^ the RGD sequence has been initially recognized in numerous ECM proteins, including fibrinogen, vitronectin, laminin, and collagen.^128–131^ Specifically, Acharya et al. investigated the expression levels of DC maturation markers and cytokine production was enhanced in response to increased RGD peptide density, with IL-12p40 being the most sensitive marker.^66,132^ To better comprehend the effect of RGD in modulating the FBR, Swartzlander et al. investigated the immune response toward polyethylene glycol (PEG), PEG-RGD and PEG-RDG hydrogels, a bioincompatible peptide with similar chemistry as RGD. An equivalent profile and quantity of adsorbed proteins among these three hydrogels was observed. Conversely, inflammatory cells can recognize the adhesion peptide RGD, and significantly reduced fibrous capsule density and thickness in comparison with PEG or PEG-RDG. Their studies further verified that RGD might influence the immunogenic cellular phenotype and function without modifying the composition of the adsorbed protein layer. A possible attribution could be the retention of the intrinsic structure of proteins on PEG-RGD, which might otherwise unfold without RGD.^123^

### Soluble danger signals released from residual cell components

DAMPs, such as HMGB1 and HSPs, not in high abundance though, were also identified.^123^ According to Babensee, a complicated mixture of DAMPs (such as heat shock proteins (HSP60), HSP70, HMGB1, and heparin sulfate), either as a consequence of injuries or an outcome of conserved material properties, was observed in the tissue adjacent to subcutaneously implanted PLGA scaffolds.^115^ It is, therefore, speculated that by binding DAMPs adsorbing to the surface of implanted biomaterial, DCs surface TLRs or CLRs might initiate biomaterial-mediated inflammation.^133^ In addition, Farshid et al. believed that the conformation of biomaterial-determined adsorbed proteins might lead to the emergence of various BAMPs, which was similar to an exogenous DAMP (including HSPs and HMGB1) and generate DC-mediated immune responses through integrins and PRRs.^134^ However, this interpretation should be viewed with caution and substantiated experimentally.

#### High mobility group box 1 (HMGB1)

The axis high mobility group box 1 (HMGB1) and the Receptor for Advanced Glycation End Products (RAGE) are related to inflammatory and healing events, with HMGB1 being the prototypical and well-characterized DAMP.^135^ Considering this, molecular dynamics computer simulations have been introduced to explore the content and conformation of HMGB1 induced at the surface of titanium.^136^ Biguetti reported that the inhibition of HMGB1 or RAGE promoted inflammatory cell infiltration, impaired osseointegration, and affected organic and mineralized bone matrix dynamics.^137^ Nevertheless, questions remain as to whether the enhanced HMGB1 detected in the implantation site is a consequence of injuries that prime the immune system, or an outcome of biomaterial intrinsic properties. To further elucidate the specific constitution, Bennewitz performed immunoblotting analysis on the tissue exudates surrounding implanted PLGA scaffolds and injected PLGA MPs.^138^ In addition to the intragroup heterogeneity arising from tissue damage which increased the concentration of DAMPs, higher levels of HMGB1 were detected around PLGA materials, signaling the potential role of HMGB1 in DC-biomaterial interactions.

#### Heat shock proteins (HSPs)

HSPs are a family of conserved chaperone molecules that prevent aggregation and misfolding of nascent polypeptides while expediting protein folding, thereby maintaining cellular functions.^139^ HSPs (e.g., HSP 70A, 70B, 9,0 and 47) expressions were modulated by the hydrophilicity of the biomaterial,^140^ which have been involved in the host immune response via regulating the expression of TLR2 and TLR4.^141^ Babensee concluded that HSPs can probably link biomaterial-mediated cell stress or necrosis and the induction of innate immunity at implant sites.^115^ Having confirmed the presence of HSP60 within the substrates, McKiel also observed increased NF-κB/AP-1-dependent SEAP activity on poly(methyl methacrylate) (PMMA) and polydimethylsiloxane (PDMS) with 10% FBS, which induced the expression of proinflammatory cytokines as compared to those preadsorbed with serum only. They speculated that inflammatory cellular function and immunogenic responses rely highly on the biomaterial-directed protein adsorption.

### Conserved structures characteristic of the material itself

Intrinsic structures characteristic of biomaterials (e.g., surface chemistry, hydrophilicity) determined inflammatory cellular interactions, in particular DCs, and the subsequential immune reactions. For instance, when an implanted hydrophobic biomaterial interact with blood, proteins may instantaneously adsorb, generally through hydrophobic interactions.^142^ In contrast, hydrophilic biomaterials indirectly generate an interfacial free energy of low value with biological fluids.^143^ Hasek uncovered multiple-layer protein adsorption profiles on the surfaces of PEG hydrogels in vivo, as a consequence of hydrophobic interactions within polyacrylate chains^144^ or through hydrogen bonding amid PEG and amide groups contained in the polypeptide mainstay of the proteins.^145^

Carbohydrates at the DC-biomaterial interface, as a key component of the adsorbed protein or an intrinsic material structures, are prominent in balancing the biocompatibility and biodegradability of biomaterials.^146^ Considering the evidenced linkage between altered glycosylation and disease progression,^147^ it is speculated that biomaterials may display analogous segments to migrating DCs if the adsorbed proteins are conformationally altered and present a modified carbohydrate profile.^148^ To confirm the existence of analogous BAMPs in the immunogenic cellular-biomaterial interface, Shankar and coworkers conducted enzyme-linked lectin assays (ELLA) to probe for carbohydrate ligands of CLRs on self-assembled monolayers (SAMs) delivering -OH, -CH_3_, -COOH or -NH_2_. They observed higher α-galactose on COOH SAMs and higher mannose on NH_2_ SAMs, indicating that unique presentation of associated carbohydrates at the biomaterial interface mediates the interactions of the innate immune response.^149,150^

To our knowledge, however, efforts to systematically characterize the molecular composition of BAMPs have just set off, and the mechanisms by which DCs recognize and react to biomaterials have not yet been fully elucidated. To better identify the adsorbed protein profiles and involved posttranslational modifications, including carbohydrates and lipids, sophisticated tools (mass spectrometry (MS),^151^ SDS-PAGE and immunoblot analysis^152^) and statistical modeling (PCA,^16,153,154^ PLSR,^155^ high-throughput nanoimmunoassay chip,^156^ single-cell analysis^157^) have been introduced. Hopefully, extensive interpretation of the immune events involved in the formation of adsorbed proteins and biomaterial recognition might provide insights for the development of next generation of biomaterials.^158^

## Mechanisms underlying dendritic cells response to biomaterials

### Integrins

Integrins refer to heterodimeric type I transmembrane proteins consisting of α and β subunits, which also constitute one of the major intracellular receptors that modulate cellular survival, proliferation, and differentiation.^1,159,160^ Random permutation of subfamilies of α and β subunits contributes to the formation of 24 distinct types of integrins, and the partnering of different subunit chains confers variable function to the receptor.^1^ Among them, leukocytes uniquely express the β2 subfamily of integrins, including αLβ2 (CD11a), αMβ2 (CD11b), αXβ2 (CD11c), and αDβ2 (CD11d). Moreover, a battery of immune events are regulated via β2 integrins: recruitment to sites of inflammation,^161^ intracellular contact^162^ and signaling.^163^ Rogers et al. observed that β2 integrins in DC adhesive podosomes are at the biomaterial interface and in direct contact with TCPs and PLGA films, and DCs treated with anti-β2 integrin antibody expressed lower levels of CD86. In this regard, their study demonstrated that a basal level of β2 signaling is a prerequisite to manipulate DC adhesion and maintain maturation in response to biomaterials.^164^ Similarly, Farshid et al. postulated that DC adhesion to the biomaterial via integrins correlated highly with DC maturation, which in turn determined the immune responses.^1^ Having reviewed published work concerning the role of DC integrin in biomaterials, Franz speculated that integrin signaling might serve as an alternative mechanism of DC activation to PRR engagement.^165^

**Fig. 4:**
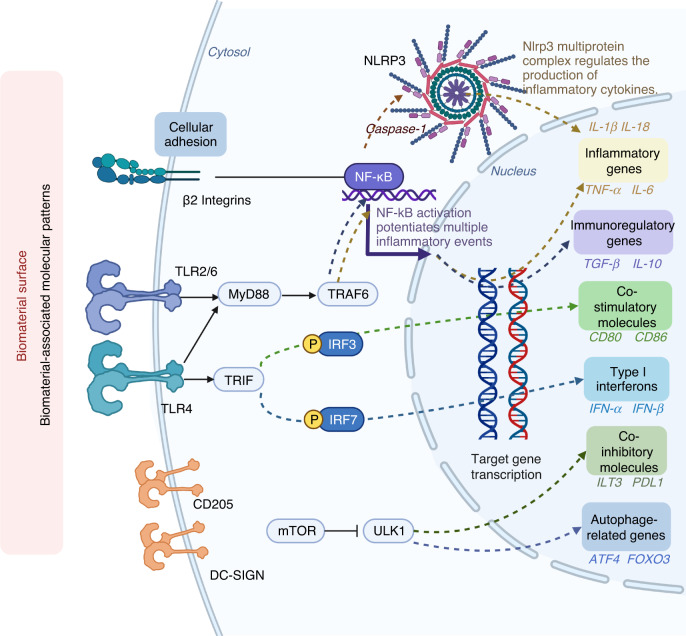
Mechanisms underlying dendritic cells response to biomaterials. **a** The mechanism of integrins. β2 integrins are virtually the first receptors adhering to the ECM proteins, thus initiating NF-kB signaling pathways, which potentiates multiple inflammatory events. **b** The mechanism of TLRs, especially of TLR 2/4/6. TLR 2/4/6 signals through MyD88 or TRIF respectively, which terminated at two events: (i) activates the NF-kB-mediated regulation of inflammatory (like TNF-α or IL-6) and immunoregulatory genes (like TGF-β or IL-10); (ii) phosphorylates IRF3 and IRF7, leading to the consequential expression of co-stimulatory molecules such as CD80 and CD86 and production of type I interferons (e.g., IFN-α and IFN-β). **c** The mechanism of CLRs. DC-SIGN and CD205 are among the most efficient CLRs that enhance antigen uptake and presentation by DCs. **d** The mechanism of inflammasomes, especially of NLRP3. In the presence of NF-kB, cytosolic NLRP3 can also be stimulated via biomaterial-derived signals and forms an intracellular multiprotein complex that catalyzes the conversion of caspase-1, thus regulating the production of highly inflammatory cytokines such as IL-1β and IL-18. **e** The mechanism of autophagy. DCs in the presence of certain biomaterials express high levels of ILT3 and PDL1, as characteristic co-inhibitory molecules of tolDC, while upregulate transcriptional profile of ATGs such as ATF4 and FOXO3

Integrins are the initial adhering receptors for ECM proteins, thus enabling the nuclear transcription factor-kappa B (NF-κB) and Jak/STAT signaling pathways,^166^ the former of which is considered a critical mediator of DC maturation molecules, including CD80 and CD86.^167^ However, the intracellular bonding between integrin engagement and DC maturation is controversial. DCs treated with PLGA films after 24 h did not necessarily reveal enhanced expression of translational NF-κB.^168^ Considering this, one possible explanation indicated by Rogers was that adhesion alone was insufficient for DC activation, which merely allowed for the localization of other coreceptors for engagement.^164^ It is reasonable, in this context, to assume coreceptors other than integrins, such as TLRs and CLRs, to be effective targets associated with DC-biomaterial interactions, the role of which will be elaborated below (Fig. 4).

### Toll-like receptors

TLRs with leucine-rich repeats in their ectodomains^169,170^ are PRRs that bind PAMPs derived from pathogens and DAMPs from dying or injured cells,^171^ the implication of which has been extended to BAMPs.^115^ Thirteen subtypes of TLRs have been reported, and among them, a retro-viral insertion has inactivated TLR10 in mice,^172^ whereas TLR11, −12, and −13 do not occur in humans.^173^ TLR1, −2, −4, −5, −6, and −10 are presented at the cell membrane, which mainly recognize microbial components, including lipids, proteins, and lipoproteins, while TLR3, −7, −8, −9, −11, −12, and −13 are expressed in the endosome.^174,175^ All TLRs signal through myeloid differentiation primary-response protein 88 (MyD88), a crucial adaptor molecule recruited upon binding of TLRs, DAMPs and BAMPs,^176^ except for TLR3, which confers to the TIR domain containing adapter-inducing interferon-beta (TRIF), and TLR4 through both MyD88 and TRIF.^177^ The network map of TLRs has been delineated substantially owing to everlasting efforts, which terminated at two events: (1) potentiates the transcription factor NF-κB^178,179^ which are involved in the regulation of many proinflammatory and immunoregulatory genes;^180,181^ (2) phosphorylates IFN regulatory factor 3 (IRF3) as well as IRF7,^1^ resulting in the consequential costimulatory molecules expression and type I interferon secretions, such as IFN-α and IFN-β.^175,182^

Apart from the large body of work exploring macrophage-induced immune responses toward orthopedic metallic implants,^177^ biomedical polymers,^183–186^ nanoparticles, and microparticles,^187^ the involvement of TLR signaling is progressively implicated in DC-biomaterial interactions. TLR4 serves as one of the most potent PRRs^188^ and is considered the core receptor in the recognition of ligands, including LPS,^189^ HSP60 and 70,^141^ HMGB1,^190^ hyaluronan,^191^ and fibrinogen.^192^ A previous investigation by Rogers and colleagues revealed that once implanted intraperitoneally in TLR4-knockout mice for 16 h, PET discs triggered an altered infiltration of leukocytes, which shifted from equivalently neutrophils and monocytes/macrophages to predominantly neutrophils, suggesting the critical role of TLR4 in the primary recognition of biomaterials.^171^ Nevertheless, no differences could be discerned in regard to the thickness of fibrous encapsulation encompassing the PET discs at 14 days, indicating that the impact of TLR4 was confined to the acute phase in response to the implanted biomaterial. In contrast, another study in C57BL6-TLR4^-/-^ mice reported less inflammatory cell infiltration and angiogenesis around the silicone prosthesis and a 1.96-fold thicker capsule around the implant in the absence of TLR4 at Day 14, pointing out the drastic effect of TLR4 in the host response to silicone implants.^193^ Between the two opposite findings, the preadsorbed fibrinogen might account for the different extent to which immune reactions are exacerbated, and it coincides with and broadens our previous postulation that the adsorbed protein layer might mediate fibrous encapsulation and the subsequent temporal immune response via TLR4.^186^ By MyD88-deficient DCs, Shokouhi et al. clarified that complicated TLR/MyD88- signaling (particularly TLR2, TLR4, and TLR6) was involved in DC-biomaterial interactions, leading to the activation and maturation of DCs and conferring on them the ability to switch on antigen-specific T cells.^31^ To further clarify the influence of TLR4/MyD88 signaling in DC-mediated biomaterial recognition, Uto et al. incubated murine BMDCs with biodegradable nanoparticles (NPs) supplemented with poly(g-glutamic acid) (g-PGA). Significant enhancement regarding costimulatory marker expression (e.g., CD40, CD80, and CD86) and cytokine release (e.g., TNF-α, IL-6, and IL-12) was detected, which were probably mediated through phosphorylated forms of p38, SAPK/JNK, and ERK. Nevertheless, consistent trends were not observed in MyD88^−/−^ or TLR4^−/−^ mice.^194^ Collectively, their work elucidated that biomaterial-triggered DCs that functioned predominantly via TLR4-MyD88-MAPK signaling pathways.

### C-type lectin receptors

CLRs strongly participate in the immune response and biomaterial recognition,^195^ representing a family of receptors, such as collectins, selectins, lymphocyte lectins, and proteoglycans.^196^ Located on the surface of DCs, CLRs ligate to the carbohydrate structures of pathogens, self-antigens and biomaterials.^115,197^ DC-specific intracellular adhesion molecule-3 grabbing nonintegrin (DC-SIGN) recognizes mannose-containing structures as well as Lewis antigens (Le(a), Le(b), Le(x), and Le(y)).^198^ By exploiting DC-SIGN ligand multivalency, internalization, lysosomal trafficking, antigen presentation and cytokine release could be optimized with glycopeptide polymers Le(b)-conjugated poly(amido amine) (PAMAM).^199^ Additionally, the notable feature of CD205 to facilitate DC antigen uptake and presentation in an exceptionally efficient and specific manner has rendered it one of the research focuses. Specifically, CD205 could be a bilateral mediator, either immunogenic or immunotolerant, depending on the maturation level and the expression of stimulatory signals.^200,201^

CLR recognition is influenced by the glycosylation of the carbohydrate recognition domain (CRD) region in dendritic cell immunoreceptor (DCIR) via its immunoreceptor tyrosine-based inhibitory motif (ITIM). Currently, researchers have been devoted to clarifying the role of CLRs in DC-biomaterial interactions and in this manner shed novel light on engineering cellular function and modulating immunity. Hotaling et al. showed that as the cationization level of conjugates or glycan density increased and so did nonspecific lectin binding. Once blocked with EDTA or specific antibodies for DC-SIGN or Dectin-1, antigen internalization and maturation of DCs was inhibited.^158,202,203^ Interestingly, the interplay of CLRs and TLRs in DCs could possibly result either in the onset of inflammatory reactions or in tolerance maintenance by the defense system.^204,205^ Nevertheless, studies concerning the collaborative role of CLRs and TLRs are rare and call for future efforts.

### Inflammasomes

Inflammasomes refer to a family of cytosolic multiprotein complexes^206^ and play a critical role in pathogen clearance, adjuvant activity, and overall immunity.^207^ Upon binding with NOD-like receptors (NLRs),^206,208^ inflammasomes function through activation of caspase-1, 4, 5, or 11 and cleavage of cytokines, including IL-1β and IL-18, followed by pyroptosis.^206^ Among the well-understood inflammasomes, NLRP3 is effective in antitumor immunity and vaccination responses induced by DCs.^209–211^ However, abnormal inflammasome activation has been associated with numerous autoimmune diseases.^212^ Therefore, some researchers have attempted to tune inflammasome-mediated immunomodulation by harnessing the properties of implanted biomaterials in an attempt to facilitate future applications in immunotherapies. Li et al. found that NLRP3 played a substantial role in mesoporous silica microrod (MSR) scaffold-induced BMDC IL-1β secretion in vitro as well as immune cell trafficking in vivo.^213^ Most DCs recruited to scaffolds in NLRP3^−/−^ mice exhibited an immature phenotype when compared to WT mice.^213^ Sharp et al. demonstrated that uptake of PLG microparticles by DCs dramatically accelerated IL-1β secretion and caspase-1 activation, which depended on the NLRP3 inflammasome, yet the production of antigen-specific antibodies was not influenced.^214^ Similarly, another study also observed that the immunogenicity of peptide nanofibers was not necessarily accompanied by the production of IL-1β or caspase-1/11. Their findings confirmed that activation of the NLRP3 inflammasome might not be a decisive factor involved in T-cell-mediated immune responses against these materials.^215^

To elucidate the underlying mechanism, previous studies reported that uptake of microparticles could induce lysosomal damage,^214^ and DAMPs generated during this process were recognized by NLRP3.^216^ Upon activation, NLRP3 forms an intracellular multiprotein complex that manipulates cytokine release (e.g., IL-1β and IL-18), leading to the infiltration of inflammatory cells for cleavage of the local particulates.^213,216^ Li et al. also considered that a similar pathway might function in terms of host immunity toward MSR scaffolds,^213^ and lysosomal perturbation might be a fundamental regulator of NLRP3 inflammasome activation.^216,217^ As Saikat et al. indicated, lysosomal rupture is an initial step in inflammasome activation, which then precedes other pathways.^217^

### mTOR-NF-kB mechanism involved in autophagy

Autophagy refers to a lysosomal pathway for cellular homeostasis control, which is presently known to be the signal light of immune cells, orchestrating their proliferation, cytokine secretion, and survival. Reactive oxygen species (ROS) serve as crucial regulators of oxidative stress and redox signaling in immune cells,^218^ the inhibition of which is involved in the tolerogenic phenotype and allo-stimulatory capacity of DCs.^101^ Nevertheless, the involvement of ROS in the manipulation of DC-mediated immunity remains to be explicitly examined, considering some opposite findings.^219^ In addition, mammalian target of rapamycin (mTOR) is a decisive mediator of cellular metabolism, NF-κB translocation, phenotypic conversion, antigen presentation, and T-cell activation of DCs.^220–222^ Autophagy is thus mediated by mTOR inhibition-induced activation of Atg1/Unc-51-like autophagy activating kinase 1 (ULK1) and subsequent posttranslational modifications of autophagy-related genes (ATGs).^223^

The role of autophagy in immunogenic function and cytokine production upon exposure to biomaterials was explored in monocytes and macrophages.^224–226^ A recent study revealed that DCs in contact with GQD presented upregulated ILT3 as well as PDL1, characteristic coinhibitory molecules of tolDCs, and were capable of potentiating allogeneic CD4^+^CD25^hi^FoxP3^+^ Treg cells. Autophagic turnover (flux) was enhanced in GQD-treated DCs, as evidenced by the upregulated transition from microtubule-related LC3-I to autophagosome-associated LC3-II and the upregulated transcriptional profile of ATGs, such as ATF4, FOXO1, and FOXO3. Most notably, genetic suppression of autophagy via RNA interference (RNAi) techniques undermined the protolerogenic effects of GQD on DCs.^101^ Consistently, the delivery of the autophagy inhibitor chloroquine enhanced p62 and LC3-II expression and reduced IL-12p70 release in human monocyte-derived DCs in a p38-dependent manner, thereby promoting Th17-mediated persistent inflammation.^227^

## Conclusions and future perspectives

Novel understanding of immune responses towards implanted biomaterials can excitingly help tackle the challenges of protracted inflammation or immune-mediated rejection, by harnessing the specificity of immune cells-biomaterial interactions. During the process, immunocytes are often recruited consecutively in an organized manner in the context of foreign biomaterials, with DCs playing an orchestrating role. Within the last decade, DC-mediated immune responses toward implanted biomaterials have been demonstrated to be diverse according to their physiochemical properties, the comprehensive interpretation of which may benefit future vaccination, cancer therapy, and the design and manufacture of novel biomedical materials. However, challenges remain. First, the new paradigm in biomaterials research calls for the interaction with the immune system instead of simply suppressing it. Considering this, incorporation with bioactive inorganic ions is universally viewed as a convenient, effective and long-lasting tool to promote desired effects. Nevertheless, their regulatory effects on DCs remain not fully elucidated. Herein, increasing research efforts should focus on incorporating available biomaterials with biological ions, in an effort to improve the effectiveness and security of their profound applications. Harnessing DC-mediated immune microenvironment for material rejection or integration is promising. Biomaterials carrying antigens or delivering vaccines can enhance host immune reactions and aid in anti-tumor therapy, while dampening immune responses by surface property modifications will assist in autoimmunity and tissue engineering. One major obstacle here in elucidating the immunogenic effects of material properties lies in its multifactorial features. To cope with this, persistent efforts should be distributed here to delineate a comprehensive map with regard to material property-DC performance interactions. Fortunately, the application of immune-mediated tissue engineering has witnessed immense growth in recent decades with the development of high-throughput strategies (e.g., PCA and PLSR). Furthermore, BAMP serve as the key immunogenic cue associated with DC-biomaterial interactions. To our knowledge, however, efforts to systematically characterize the molecular composition of BAMPs have just set off, and the mechanisms by which DCs recognize and react to biomaterials have not yet been fully elucidated. To extensively identify the adsorbed protein profiles and involved posttranslational modifications, including carbohydrates and lipids, sophisticated tools and statistical modeling have been introduced, which might provide insights for the development of next generation of biomaterials. In addition, spatiotemporal activation of dendritic cells and their precise modulation mechanism can provide insights for future development of novel biomaterials, and such approaches have not been well explored in the tissue engineering community. It is postulated that multiple receptors might be associated with DC-biomaterial interactions collaboratively. Better understanding towards the underlying mechanisms could pave the way for the rational design and clinical application of material-based strategies with desired immune effects. Notably, the immunologic microenvironment is rather complex and rapidly changeable, while the majority of the work in this field relying on in vitro studies is less convincing. Compounding this, the remarkable advances in cell biology and biomaterials science and engineering also add to the uncertainty and complexity. Therefore, much work still needs to be done to unveil the behavior and biofunction of DCs toward novel implanted biomaterials in vivo.

## Supplementary information

Supplementary Figure S1
