# Supplementary material for: The role of dendritic cells in the immunomodulation to implanted biomaterials
Source: Int J Oral Sci. 2022 Nov 4;14:52. doi: 10.1038/s41368-022-00203-2 (PMC9636170; doi:10.1038/s41368-022-00203-2)
Supplement: Supplementary file 1 — Supplementary Figure S1 [file 41368_2022_203_MOESM1_ESM.docx]

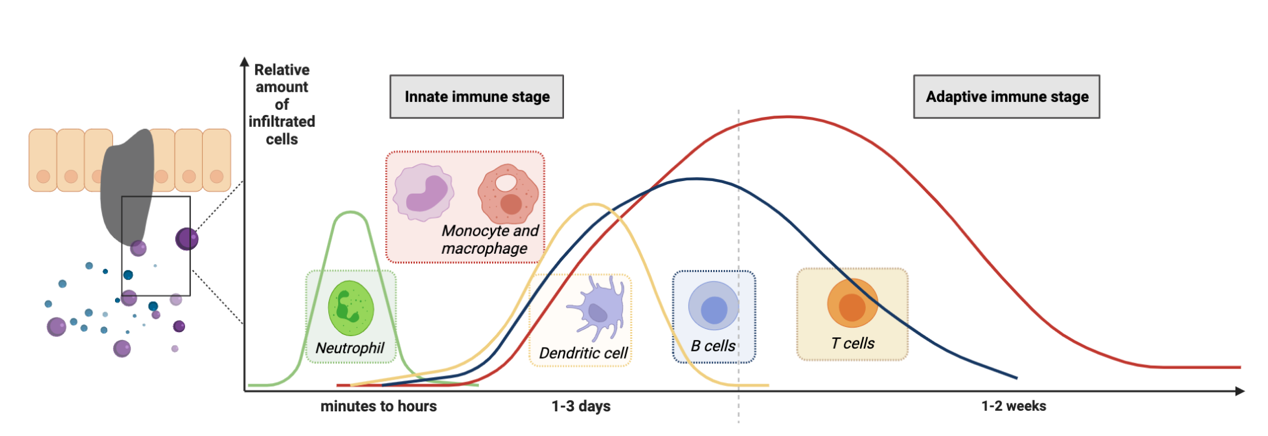


**Supplementary Figure S1** Type and amount of cells recruited to the implant sites. Host proteins majorly adhere to the surface of biomaterials within 4 hours, which are followed by a series of subsequential chain responses like the infiltration of immune cells and secretion of cytokines and chemokines. As such, neutrophils, monocytes, macrophages, DCs, B and T cells are recruited to the biomaterial interface consecutively. Specifically according to Anderson and James^34^, neutrophils have short lifetimes of hours to days, while monocytes rapidly differentiate into macrophages, which have lifetimes of days to weeks to months. Notably, a delayed increasing B cell beyond day 7 was observed towards implanted alginate^35^, whereas our unpublished work has identified early onset of B cells at the peri-material milieu in mice tibiae (15.78%, day3). Physiochemical properties and biocompatibility of the implanted biomaterials may be explicative for variations in the intensity and time duration of the inflammatory processes.

**Supplementary Table S1 The effects of physiochemical properties on DC phenotype and functions**

| **Physiochemical properties** | **Biomaterials** | **Surface generation methods** | **DC origins** | **DC morphology** | **DC phenotype** |
| --- | --- | --- | --- | --- | --- |
| **Chemical composition** | alginate, agarose, chitosan, hyaluronic acid (HA), PLGA (75:25) | Films are formed by casting discs into a Teflon Petri dish | DCs derived from human peripheral blood monocytes | DCs cultivated on chitosan and PLGA films exhibited maturation morphology. | DCs cultured on chitosan and PLGA films (agarose to a lower level) expressed higher levels of CD40, CD86, and HLA-DQ in DCs; whereas HA and alginate reduced their expressions. |
|  | agarose, PLGA (75:25) | PLGA MPs: emulsion solvent evaporation technique; PLGA films: casting technique without a porogen; Agarose MPs: polystyrene MPs coated with agarose of 2-30 um in diameter; Agarose films: dispense 1mL agarose solution and solidify at 48℃ for at least 30 min | human monocyte-derived DCs | DCs treated with agarose and PLGA MPs and films showed similar dendritic processes. | DCs cultured on PLGA film expressed increased levels of CD80, CD83, CD86, HLA-DQ and HLA-DR compared to those on agarose films or MPs. |
|  | PLGA (75:25) | PLGA MPs: emulsion solvent evaporation technique; PLGA films: casting technique without a porogen. | BMDC from 5 to 7weak-old male C57BL6 mice | DCs cultivated with PLGA MPs or films displayed dendritic processes to a degree between those of iDCs and LPS-induced mDCs. | DCs expressed increased CD80 and CD86 after the cultivation on PLGA MPs or PLGA film. |
|  | alginate, agarose, chitosan, hyaluronic acid (HA), PLGA (75:25) | Films are fabricated by a modified casting technique. | DCs derived from human peripheral blood monocytes | DCs cultured on PLGA or chitosan films showed dendritic processes comparable to mDCs, conversely those with alginate, agarose or HA films were similar to iDCs without processes. | Cultivation with PLGA or chitosan films upregulated DCs expression of CD80, CD83, CD86, and HLA-DQ than iDCs, so did those treated with alginate except for CD80. However, treatment with HA films significantly reduced co-stimulators expression. |
|  | alginate, chitosan, agarose, PLGA (75:25), hyaluronic acid (HA), | Films are established by a modified technique using a Teflon Petri dish | DCs derived from human peripheral blood monocytes | Not mentioned. | Not mentioned. |
| **Surface chemistry** | Ca^2+^, Ba^2+^-alginate gel, agarose, collagen gels, TCP | thoroughly mixed CaSO_4_ or BaCl_2_ is slurry quickly pipetted and stirred into the alginate and cured | BMDC from C57BL/6 mice aged 4~16 weeks | Ca^2+^ enhanced stimulatory and co-stimulatory molecules (e.g., CD86 and MHC class II) expression in DCs. | Ca^2+^ upregulated inflammatory IL-1β secretion |
|  | γ-AlOOH mesostrands incorporated with Zn^2+,^ Cu^2+^ buds | CuCl_2_ and AlCl_3_ mixed in water and incubated in shaking bath at 40℃ for 24h | BMDC from femurs of mice | Both Zn^2+^ and Cu^2+^ enhanced early expression of CD86 | Both Zn^2+^ and Cu^2+^ increased the secretion of INF-γ cytokine. |
|  | Zn^2+^, Cu^2+^ bioactive glasses | analytical grade reagents melted, annealed, crushed, and sieved to obtain a particle size in the range of 300-500um | BMDC from C57BL/6 mice | Cu^2+^ reduced expression of surface marker like MHCII, CD80, CD86 and CD25, whereas Zn^2+^ promoted expression of CD11c, CD80 and CD86. | Cu^2+^ increased secretions of IL-1, IL-17A and IL-6 in DCs. |
| **Hydrophilicity** | PLA，PLGA，PELA | premix membrane emulsification | BMDC from female BALB/c mice | Not mentioned. | PLA induced higher expression level of CD86 and MHC II in DCs when compared with PLGA or PELA. |
|  | Clinical Ti discs including PT, SLA, and modSLA; TCP controls | 15 mm diameter Ti disks | DCs derived from human peripheral blood monocytes | PT, SLA increased expression of CD86 in DCs, whereas modSLA and TCP controls induced immature morphology with decreased expression of CD86 | Not mentioned. |
|  | Clinical Ti discs including PT, SLA, and modSLA; TCP controls | Grade 2 unalloyed Ti disks (1 mm in thickness and 14 mm in diameter) | murine-derived BMDC | PT, SLA increased expression of CD86 in DCs while modSLA and TCP controls induced immature morphology with decreased expression of CD86 | Not mentioned. |
|  | Classical tissue culture plates, Clinical Ti discs including PT and SLA | 15 mm diameter discs supplied by Straumann AG (Basel, Switzerland) | DC2.4, immature dendritic cell line derived from murine myeloid (Bioshhy, China) | PT, SLA induced highly dendritic morphology. | PT, SLA induced higher CD86 fluorescence intensity. |
| **Spatial structures** | 3D collagen-chitosan nanoscaffolds, 2D culture plates | chitosan solution dissolved in 1% acetic acid, dripped into collagen suspension and homogenized | DCs derived from bone marrow of BALB/c mice | Not mentioned. | DCs treated on 3D collagen-chitosan scaffolds increased MHC II, CD40 and CD86 expression. |
|  | 3D pHEMA and PDMS of three pore sizes, including20, 40, and 90 um | patented sphere-templating method | JAWSII murine dendritic cell line, resembling LPS-mDC | 20um-pore-scaffolds promoted the dendritic morphology of DCs. | 20um-pore-scaffolds promoted CD86 and MHC II expression, in spite of the polymer used. |
|  | 2D culture plates and 3D scaffolds | 3D collagen matrices | THP-1 human monocytic cell line- based DC-like model | Not mentioned. | Surface markers like CD86 were up-regulated in 2D culture, whereas they were regulated by matrix density in 3D culture. |
| **Surface charge** | large GQD: -9.0 ± 1.5 mV; small GQD: -9.4 ± 0.8 mV | GQD fabricated by electrochemical procedure, with spectroscopic graphite rods as anode and cathode. | peripheral blood mononuclear cells | Not mentioned. | DC matured with GQD displayed a tolerogenic phenotype with down-regulated expression of CD40, CD83, CD86, and HLA-DR. |

**Supplementary Table S1** (continued)

| **Physiochemical properties** | **DC functions** | **Effects on other immune cell responses** | **Effects on non-immune cell responses** | **Year, Author** | **Ref** |
| --- | --- | --- | --- | --- | --- |
| **Chemical composition** | Not mentioned. | Not mentioned. | Not mentioned. | 2005 Babensee | 62 |
|  | DCs cultured on PLGA films stimulated allogeneic T cell proliferation, instead of those seeded on agarose films. Treatment with PLGA films upregulated production of TNF-α and autocrine factor than iDCs. Moreover, DCs cultured with agarose or PLGA film (agarose to a higher level) exhibited NF-kB activation level between those of LPS- matured DCs and iDCs. | Not mentioned. | Not mentioned. | 2006 Yoshida | 63 |
|  | DCs cultivated on PLGA MPs and films produced more proinflammatory cytokines TNF-αand IL-6 in DCs, wherein MPs induced higher expression level of TNF-α than did films | Not mentioned. | Not mentioned. | 2007 Yoshida | 44 |
|  | DCs cultivated on PLGA or chitosan films induced T cell proliferation in contrast to iDCs, whereas DCs cultivated on HA films inhibited T cell proliferation. Cultivation on PLGA, alginate or chitosan (chitosan highest) films upregulated DCs cytokine release (e.g., TNF-α) in comparison with iDCs, whereas those cultured on HA films secreted less TNF-α. | Not mentioned. | Not mentioned. | 2012 Park | 61 |
|  | DCs cultured on agarose films secreted higher levels of IL-12p70, so did those seeded on chitosan films, while those treated with agarose films presented lower expression profiles in contrast to iDCs. In addition, cultivation with agarose films induced lower levels of IL-4 expression. | DCs cultured on agarose or alginate films stimulated CD4^+^CD8^+^ T cells to a significantly larger extent, and those treated with agarose films resulted in upregulated expression of CD4^+^CD25^+^ in CD3^+^ T cells. DCs seeded on agarose films upregulated expression of FoxP3 in CD4^+^CD25^+^ T cells compared to those treated with PLGA films. | Not mentioned. | 2015 Park | 97 |
| **Surface chemistry** | Ca^2+^ upregulated inflammatory IL-1β secretion | Not mentioned. | Not mentioned. | 2013 Chan | 73 |
|  | Both Zn^2+^ and Cu^2+^ increased the secretion of INF-γ cytokine. | Both Cu^2+^and Zn^2+^ enhanced population of CD4^+^CD8^+^ T cells in spleen. | Not mentioned. | 2017 Li | 75 |
|  | Cu^2+^ promoted the release of IL-1, IL-6 and IL-17Ain DCs. | Zn^2+^ promoted T cell proliferation and differentiation into Th17, while Cu^2+^ inhibited T-cell proliferation or Th17 differentiation, meanwhile induced a down-regulated cytokine profile in T cells. | Not mentioned. | 2020 Schuhladen | 74 |
| **Hydrophilicity** | Not mentioned. | Increased antigen trafficking to LNs and upregulated Teff (T cell- mediated cytokine secretion) was observed in DCs treated with PLA. | Not mentioned. | 2013 Liu | 82 |
|  | Not mentioned. | Not mentioned. | Not mentioned. | 2011 Kou | 16 |
|  | Not mentioned. | Not mentioned. | modSLA-DC-CM promoted osteoblast differentiation by increasing the expression of RUNX2, ALP; increased ALP activity | 2017 Zheng | 17 |
|  | PT, SLA increased release of proinflammatory IL-6, IL-12, IL-18 in DCs, whereas TNF-α, IL-1ra, IL- 4, and IL-10 were reduced. | Not mentioned. | PT, SLA-DC-CM down-regulated the expression of COL1, RUNX2, OCN, and ALP; reduced ALP activity and mineralization nodules | 2019 Yang | 18 |
| **Spatial structures** | Upregulation of IL-12, IL-6 and TNF-a by DCs were observed on 3D collagen-chitosan scaffolds. | DCs stimulation of T cells IFN-γ and IL-4 release was promoted in the 3D system, while TGF-β1 secretion was inhibited. | Not mentioned. | 2016 Daneshmandi | 88 |
|  | TNF-a, MIP-1a andIL-6 secretion by DC were enhanced on 20um-pore-scaffolds, regardless of the polymer used. | Not mentioned. | Not mentioned. | 2018 Chen | 84 |
|  | Cytokines are generally increased in 2D culture, whereas in 3D culture they are regulated by matrix density. | Not mentioned. | Not mentioned. | 2020 Sapudum | 91 |
| **Surface charge** | Not mentioned. | Cationic GQD-treated DCs inhibited the proliferation of allogeneic T cells, decrease Th1 and Th17 polarization yet increase Th2 polarization | Not mentioned. | 2017 Tomic | 101 |
